# Emerging Contribution of PancRNAs in Cancer

**DOI:** 10.3390/cancers12082035

**Published:** 2020-07-24

**Authors:** Neri Mercatelli, Ramona Palombo, Maria Paola Paronetto

**Affiliations:** 1 Laboratory of Cellular and Molecular Neurobiology, IRCCS Fondazione Santa Lucia, 00143 Rome, Italy; neri.mercatelli@gmail.com (N.M.); r.palombo@hsantalucia.it (R.P.); 2 Department of Movement, Human and Health Sciences, Università degli Studi di Roma “Foro Italico”, Piazza Lauro de Bosis, 15, 00135 Rome, Italy

**Keywords:** pancRNA 1, cancer 2, RNA binding proteins (RBPs) 3, cell cycle 4

## Abstract

“Cancer” includes a heterogeneous group of diseases characterized by abnormal growth beyond natural boundaries. Neoplastic transformation of cells is orchestrated by multiple molecular players, including oncogenic transcription factors, epigenetic modifiers, RNA binding proteins, and coding and noncoding transcripts. The use of computational methods for global and quantitative analysis of RNA processing regulation provides new insights into the genomic and epigenomic features of the cancer transcriptome. In particular, noncoding RNAs are emerging as key molecular players in oncogenesis. Among them, the promoter-associated noncoding RNAs (pancRNAs) are noncoding transcripts acting in *cis* to regulate their host genes, including tumor suppressors and oncogenes. In this review, we will illustrate the role played by pancRNAs in cancer biology and will discuss the latest findings that connect pancRNAs with cancer risk and progression. The molecular mechanisms involved in the function of pancRNAs may open the path to novel therapeutic opportunities, thus expanding the repertoire of targets to be tested as anticancer agents in the near future.

## 1. Introduction

Tumor formation is a multistep process characterized by transformation of the cell from a physiological to a neoplastic stage. During this process, cells lose checkpoints that control their growth in response to internal and external cues and acquire oncogenic features that allow them to evade growth-inhibitory signals, to invade neighboring tissues, and to colonize distant new sites by promoting neo-angiogenesis and by escaping cell death [1]. Tumor cells take advantage of a complex network of protein and RNA molecules to accomplish this multifaceted oncogenic program [1].

The extensive implementation of high-throughput sequencing technologies has revealed that less than 2% of the genome encodes proteins whereas 75% is actively transcribed into noncoding RNAs [2,3]. Excluding microRNAs, most of noncoding transcripts exceed 200 nucleotides in length and are collectively indicated as long noncoding RNAs (lncRNAs). Most of them are transcribed by RNA polymerase II, capped, and polyadenylated at their 5′ and 3′ ends, respectively [4].

LncRNAs are evolutionarily less conserved than protein-coding transcripts [5] and display tissue-specific patterns [6]. However, the function of the vast majority of them has not been elucidated yet. LncRNAs are finely regulated and their expression often correlates with genes influencing cell cycle regulation, survival, immune response, or pluripotency. Remarkably, several lncRNAs are transcriptionally regulated by key tumor suppressors and oncogenes, thus acting as effectors of the oncogenic program [7,8]. Genome-wide analyses revealed several functional cancer-associated mutations within the noncoding genome [9]. LncRNAs can drive oncogenic phenotypes through their interactions with other cellular macromolecules including DNA, protein, and RNA.

New computation methods for transcriptome analyses allowed the identification of extensive transcription of noncoding transcripts originating from active enhancers, promoters, and intergenic regions [3,10]. In particular, all eukaryotic species have been shown to transcribe noncoding RNAs from the promoter regions proximal to the transcription start-sites (TSS) [11,12,13,14,15,16,17]. These transcripts, named promoter-associated noncoding RNAs (pancRNAs), operate as *cis*-acting elements and regulate transcription of their host genes. PancRNAs display tissue specificity and contribute to many biological processes [18]. They often act as scaffolds for enzymes that modulate the epigenetic signature of promoter sequences and elicit an impact on gene expression [19]. On the other hand, alterations induced by pancRNAs have been linked to the gene expression changes occurring in human cancer [19].

**Figure 1:**
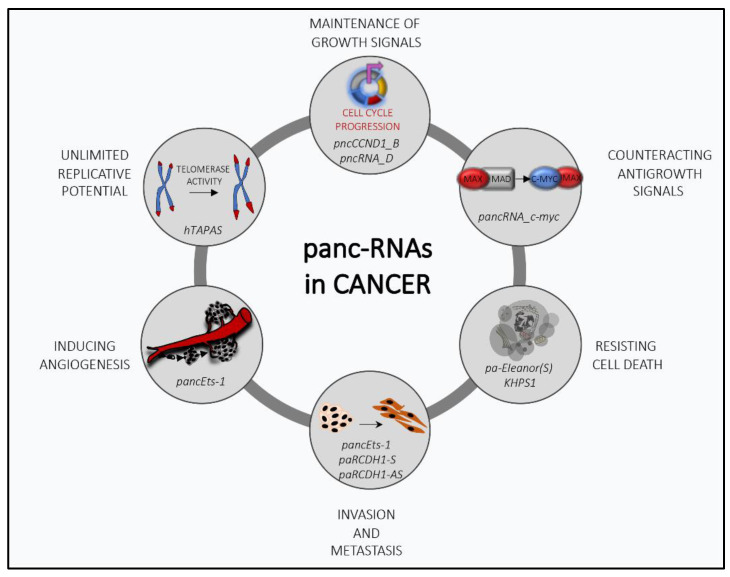
The multifaceted role of promoter-associated noncoding RNAs (pancRNAs) in cancer transformation: PancRNAs can regulate several cancer-related processes by modulating the transcriptional expression of genes involved in tumor progression.

Recent studies have revealed important details about the epigenetic dynamics of promoter-associated transcripts. These findings help establish some general regulatory characteristics of pancRNA network (Figure 1). Advancements in the comprehension of the molecular mechanisms involved in the function of lncRNAs have recently allowed drawing detailed road maps to understand their biological properties and have suggested their potential exploitation for therapeutic intervention. In this review, we will illustrate the contribution of pancRNAs to cancer biology and will discuss the latest findings that connect pancRNAs with cancer risk and progression. Finally, we will introduce new strategies that focus on pancRNAs as novel targets for therapeutic treatment.

## 2. Role of PancRNAs in Neoplastic Transformation

### 2.1. PancRNAs Support Cell Proliferation

Normal cells require mitogenic signals to switch from a quiescent condition to a proliferative state. On the contrary, cancer cells harbor mutations in oncogenes that constitutively activate mitogenic signaling pathways and/or acquire the ability to synthesize growth factors by themselves, thus establishing a positive feedback loop indicated as autocrine stimulation [1].

The identification of specific ncRNAs arising from the promoter region of the Cyclin D1 (*CCND1)* gene has paved the way to efforts aimed at understanding the putative role for pancRNAs as cell cycle regulators [20,21]. Wang and colleagues identified four pancRNAs encoded by sequences embedded in the genomic region spanning from −2008bp to −162bp of the CCND1 transcription start site (TSS) [21]. The *CCND1* pancRNAs display a specific and coordinated expression. The most characterized of them are *pncRNA-D* and *pncCCND1_B*. PncRNA-D is induced upon ionizing radiation (IR) and osmotic stress in HeLa cells, and *pncCCND1_B* recently emerged as a key molecular player in Ewing sarcoma [21,22]. Although both negatively impact cyclin D1 expression, their mechanism of action is different, mainly due to the specificity of their molecular interactors. From this point of view, the identification of RNA binding proteins (RBPs) interacting with them appears as a critical point to unravel the alternative molecular mechanisms involved in the function of pancRNAs that converge on a specific process, such as those linked to cyclin D1 regulation.

In the last decade, efforts made by the group of Prof. Kurokawa led to the identification of the RBP FUS/TLS (fused in sarcoma/translocated in liposarcoma) as a key mediator of the activity of *pncRNA-D* in the regulation of cyclin D1 [21]. FUS/TLS belongs to the FET (FUS, EWS, TAF15) family proteins and is involved in several aspects of DNA/RNA metabolism such as DNA repair, alternative splicing, and transcription [23]. The molecular complex composed by FUS/TLS and *pncRNA-D* inhibits the transcriptional activity of the *CCND1* promoter by binding to the histone acetylase CREB binding protein (CBP)/p300. Mechanistically, *pncRNA-D* induces an allosteric modification of FUS/TLS, allowing the interaction between its N-terminus and CPB/p300 [21]. Among the pancRNAs of the *CCND1* gene, *pncRNA-D* displays the highest induction after double-stranded break (DSB)-inducing treatments such as irradiation and osmotic stress in HeLa cells. By recruiting FUS/TLS to the *CCND1* promoter, *pncRNA-D* promotes a repressive epigenetic state resulting in the reduction of cyclin D1 expression. Accordingly, the inhibition of *pncRNA-D* by siRNA strategy or RNAseT1 treatment impairs the binding of FUS/TLS to the *CCND1* promoter, further supporting the dependence of FUS/TLS from this specific ncRNA in the regulation of *CCND1*. Intriguingly, the methylation status of FUS/TLS as well as of *pncRNA-D* can affect the functionality of this molecular mechanism [24]. FUS/TLS contains two glycine-arginine rich (RGG) domains in its C-terminal region, which are directly involved in the binding to pncRNA-D [25]. Methylation of arginine R-476 disrupts the pncRNA-D-FUS/TLS interaction, thus leading to cyclin D1 upregulation through the rescue of CBP/p300 histone acetyl-transferase (HAT) activity [26]. Furthermore, pncRNA-D is susceptible to m6A methylation and its methylation was dramatically decreased after osmotic stress or irradiation in HeLa cells [24]. Notably, it has been reported that the inhibition of methylation enhances the stability of *pncRNA-D*, thus promoting its interaction with FUS/TLS [24]. Therefore, the methylation process seems to ensure the control of cyclin D1 levels through two alternative ways. In line with this, strategies aimed at reducing the methylation status of both FUS/TLS and *pncRNA-D*, respectively, on arginine and adenine may reinforce their interaction and may open new perspectives for cyclin D1-based anticancer therapies.

*PncCCND1_B* is the prominent *CCND1* pancRNA in Ewing sarcoma, one of the most aggressive pediatric cancers. Ewing sarcoma is characterized by in-frame chromosomal translocation leading to the expression of the aberrant transcription factor EWS-FLI1 [27]. By binding to the RNA helicase DHX9, EWS-FLI1 promotes the transcription of oncogenes involved in Ewing sarcoma progression, including cyclin D1 [28,29,30,31]. Differently from *pncRNA-D*, *pncCCND1_B* does not interact with FUS/TLS [22]. However, it binds Sam68, an RBP that was previously shown to regulate cyclin D1 expression and alternative splicing in prostate cancer [32,33,34]. The interaction between Sam68 and *pncCCND1_B* is a key molecular event driving cyclin D1 repression in Ewing sarcoma. Particularly, *pncCCND1_B* decreases the rate of lysine acetylation of the histone H3 in the *CCND1* promoter region whereas Sam68 is necessary for the chromatin localization of *pncCCND1_B*. Sam68 forms a multimolecular complex composed by *pncCCND1_B* and the RNA/DNA helicase DXH9 in Ewing sarcoma cells. This complex is promoted by the small molecule YK-4-279, which disrupts the interaction between EWS-FLI1 and DHX9. On the other hand, mitogenic stimulation of Ewing sarcoma cells by treatment with Insulin-like Growth Factor (IGF)-1 inhibits the interaction between Sam68 and *pncCCND1_B* and leads to an increase in cyclin D1 transcription [22]. Mechanistically, IGF-1 enhances the tyrosine phosphorylation of Sam68, thus lowering its RNA binding affinity. In this condition, DHX9 is released and becomes available for interaction with EWS-FLI1, thus improving the transcriptional activity of the oncogene. Thus, the regulation of cyclin D1 expression in Ewing sarcoma cells results from the mutually exclusive binding of two different complexes to the *CCND1* promoter: one composed by EWS-FLI1 and DHX9, promoting cyclin D1 expression, and a repressive complex formed by Sam68, DHX9, and *pncCCND1_B* [19,22].

### 2.2. PancRNAs Counteract Antiproliferative Signals

In normal tissues, multiple antiproliferative signals are engaged to guarantee maintenance of tissue homeostasis and to prevent aberrant proliferation. Depending on the external environment, cells can either force cell cycle exit to reach the quiescent (G0) state or can enter into a postmitotic state, characterized by specific differentiation features [1]. One strategy exploited by cancer cells to escape from the differentiation program involves the upregulation of the MYC oncogene. The *MYC* gene encodes a transcription factor that plays multiple roles in cancer transformation. By recruiting histone-modifying and chromatin-remodeling enzymes, MYC regulates a large group of mammalian genes involved in cell-cycle regulation, protein biosynthesis, and metabolic pathways [35].

During normal development, the growth-stimulating activity of MYC, in association with MAX (MYC Associated Factor X), is replaced by differentiation-inducing alternative complexes of MAX with the MAD (MAX Dimerization Protein 1) transcription factor [36]. Overexpression of the MYC oncoprotein in tumor cells can reverse this balance by favoring the MYC–MAX complex, thereby promoting aberrant cell growth [35]. In line with this notion, transcriptional silencing of MYC was shown as an effective strategy to block maintenance and proliferation of prostate cancer stem cells both in cell cultures and in tumor xenografts [37]. In particular, *MYC* silencing was associated with reduced self-renewal and tumor-initiating capability [37]. Interestingly, transcription of low copy transcripts in the region arising from −400 to +120 of the *MYC* TSS was reported in prostate cancer cells [38]. These pancRNAs in the *MYC* promoter could play a direct role in maintaining promoter accessibility to transcription factors and in assisting them in transcription initiation. They seem to act as a trail for RNA polymerase II and core transcriptional regulators, in particular, TFIIB (Transcription Factor II B), to assemble the preinitiation complex and to start *MYC* transcription correctly. It has been shown that these pancRNAs favor the recruitment of siRNAs, forming a complex able to switch off transcription [38]. The proposed mechanism of inhibition involves the formation of a siRNA–pancRNA complex with the participation of AGO2 (Argonaute RISC Catalytic Component 2). This complex reduces the assembly of the functional pre-initiation complex and inhibits transcription initiation from the *MYC* promoter. Transcription inhibition is elicited by the disruption of the interaction between the pancRNA and the RNA polymerase II rather than by degradation of the pancRNAs [38]. This RNA-based transcriptional regulatory network engaged on the *MYC* transcription start site represents a sophisticated example of pancRNA-assisted gene expression to sustain the transcriptional machinery during cell adaptation and transformation.

### 2.3. PancRNAs Favor Apoptosis Escaping

Acquired resistance to apoptosis is a hallmark of most types of cancer. Cancer cells adapt to the surrounding environment and often develop resistance to anticancer treatments. The involvement of pancRNAs in the escape from apoptosis is exemplified by *pa-Eleanor* in breast cancer. Breast cancers expressing estrogen receptor-α (ER) depend on estrogen for cellular growth and survival. Endocrine therapies, such as aromatase inhibition to block estrogen production, are the most effective treatments for ER-positive breast cancers [39]. However, although initially responsive, most breast tumors develop resistance to therapies [39]. The MCF7 human breast cancer cells are ER-positive but can acquire estrogen-independent proliferation properties if cultured in estrogen-depleted condition, named long-term estrogen deprivation (LTED) [40]. It has been shown that the gene-encoding ER (*ESR1*) is upregulated during adaptation to LTED [41]. Such upregulation is partially due to cis-acting transcripts that are bidirectionally transcribed from the *ESR1* promoter. The adaptation process is epigenetically supported by noncoding RNAs transcribed from a large chromatin domain of approximately 700 kb, named *Eleanors* (*ESR1 locus* enhancing and ncRNAs activating noncoding RNAs) and including *ESR1* and other co-regulated genes. *Eleanor* transcripts form a RNA cloud that activates in cis transcription [42] and helps cells to withstand the hostile environment imposed by LTED. In addition to the *Eleanor* cloud, RNA-sequencing experiments revealed the transcription of a sense pancRNA named *pa-Eleanor(S*), for which expression is induced by LTED and repressed by resveratrol treatment [43]. The proposed scenario is that, in the LTED condition, pa-Eleanor(S) promotes *Eleanors* synthesis and formation of the cloud, thus leading to the activation of the neighboring genes involved in cell proliferation and apoptosis, such as *ESR1* and *FOXO3* (Forkhead box O3). The inhibition of *Eleanors* by resveratrol treatment as well as pa-Eleanor(S) knockdown affects the formation of the cloud and represses *ESR1* gene expression, whereas *FOXO3* expression is maintained.

Another example is represented by the pancRNA *Khps1*, which is functionally involved in the resistance to the E2F1-mediated apoptosis. *Khps1* acts through a finely regulated epigenetic mechanism impinging on the Sphingosine Kinase 1 (*SPHK1*) oncogene [44]. The *locus* encompassing both *KHPS1* and *SPHK1* genes shows an intricated genomic structure. *Khps1* is transcribed in the antisense orientation to the isoform B of the SPHK1 gene, for which the promoter contains a binding site for the transcription factor E2F1 and two triplex forming regions (TFRs) involved in the formation of RNA-DNA triplexes [45]. The TSS of *Khps1* is mapped in the first intron of *SPHK1-B*, and the *Khps1* coding unit covers the whole region spanning the exon and the promoter of *SPHK1-B*. Notably, transcription of *Khps1* is enhanced by the binding of E2F1 to a consensus site located upstream of the *Khps1* TSS. Once activated, *Khps1* associates with the distal TFR via formation of the RNA-DNA triplex and guides the specific recruitment of the histone acetyltransferase p300/CBP on the *SPHK1* promoter. As a consequence, histone hyperacetylation establishes an open chromatin structure which promotes the E2F1-dependent transcriptional activation of SPHK1. Interestingly, it has been documented that the SPHK1 protein is oncogenic and promotes the resistance of cancer cells to apoptosis [46]. In line with this observation, depletion of *Khps1* renders tumor cells more sensitive to tamoxifen-induced apoptosis, suggesting its therapeutic potential for the implementation of anticancer strategies [44].

### 2.4. PancRNAs Favor Unlimited Replicative Potential

Mammalian cells carry an intrinsic cell-autonomous program that limits their multiplication. This program appears to operate independently of the cell-to-cell signaling pathways [1]. Telomere length and telomerase activity contribute to the control of replicative potential and are involved in the physiopathology of cancer [47]. Several diseases associated with ageing, including cancer, are characterized by short telomeres [47]. Telomere shortening and the absence of telomerase in normal tissues counteract the transformation process and limit the number of replicative cycles. By contrast, tumor cells prevent telomere loss by aberrantly upregulating telomerase [47,48]. Telomerase activity is strictly dependent on the availability of the telomerase reverse transcriptase (hTERT) and the enzymatic component of telomerase, and expression of the this enzyme is tightly regulated at the transcriptional level through epigenetic modifications in the promoter region [49]. In line with its regulatory role, recurrent mutations in the promoter region of hTERT are among the most common somatic mutations in many types of cancer, including melanomas, glioblastoma multiforme, hepatocellular carcinomas, and bladder cancers [50,51,52].

Bioinformatic analysis of RNA sequencing data from the Encyclopedia of DNA Elements (ENCODE) consortium revealed the presence of an antisense lncRNA in the *hTERT* promoter region, named *hTAPAS* (hTERT Antisense Promoter-Associated RNA), which was also experimentally confirmed in tumor cell lines and in TGCA (The Cancer Genome Atlas) datasets [53]. hTAPAS functions as a negative regulator of hTERT expression. Overexpression of *hTAPAS* pancRNA downregulates *hTERT*, whereas its knockdown induces the upregulation of hTERT expression [53]. These findings open the hypothesis that *hTAPAS* might recruit the epigenetic machinery to regulate hTERT expression. However, *hTAPAS* is not expressed in normal tissues of cancer patients and it does not appear to be involved in the regulation of hTERT expression in somatic cells. Therefore, *hTAPAS* might contribute to the maintenance of hTERT expression exclusively in the narrow range required for telomere length homeostasis in cancer cells and stem cells.

Another transcription factor involved in cancer progression is *FOXC1* (Forkhead box C1), a key regulator of diverse cellular functions [54]. It belongs to the fork-head transcription-factor family and is abnormally upregulated in various malignant tumors [54]. A lncRNA transcribed from the upstream region of the *FOXC1* promoter, named *FOXCUT* (*FOXC1* Upstream Transcript), associates with its adjacent mRNAs in “lncRNA–mRNA pairs” and takes part in the regulatory network driving cancer progression [55,56,57,58]. However, the molecular mechanism underlying this regulation has not been elucidated yet.

### 2.5. PancRNAs Sustain Angiogenesis

Tumor growth is strictly dependent on the process of vascularization [1]. Oxygen and nutrients supplied by the vasculature are crucial for cell function and survival. Physiologically, once a tissue is formed, the growth of new blood vessels is transitory and carefully regulated. Tumors activate neo-angiogenesis by altering the balance between angiogenetic inducers and inhibitors [59]. One common strategy for shifting such a balance involves altered gene transcription. The ETS transcription factor ERG is a regulator of endothelial function [59] and plays crucial roles in promoting angiogenesis during development. In the mature vasculature, ERG maintains endothelial homeostasis [59]. Notably, its ectopic expression in non-endothelial tissues can contribute to oncogenesis [60].

*PancEts-1* is transcribed from the promoter region of the *ETS-1* gene and is associated with poor survival in gastric cancer patients [61]. *PancEts-1* interacts with the non-POU domain containing octamer binding (NONO), a multifunctional RNA/DNA-binding protein that participates in various biological processes, including transcriptional regulation, RNA processing, and DNA repair [62]. The interaction between NONO and *panc-Ets-1* promotes ERG transactivation on the *Ets-1* promoter, thus supporting the aberrant growth of gastric cancer cells [61]. The recruitment of a multimolecular complex composed by *pancEts-1*/NONO/ERG on the *Ets-1* gene drives ETS-1 expression and contributes to tumorigenesis and gastric cancer progression. Due to its strong impact on gastric cancer malignancy, this novel molecular mechanism of regulation could be further exploited for its potential therapeutic value as a novel therapeutic target for this disease.

### 2.6. PancRNAs Contribute to Tissue Invasion and Metastasis

During the development of human cancer, primary tumor masses spawn pioneer cells that move out, invade adjacent tissues, and travel to distant sites where they may succeed in founding new colonies and in generating metastases, which are the cause of 90% of human cancer deaths [1]. The capability for invasion and metastasis enables cancer cells to escape the primary tumor mass and to colonize new territories in the body where nutrients and space are not limiting [63].

The epithelial-to-mesenchymal transition (EMT) occurs both in physiological and in pathological condition and involves the disruption of cell–cell adhesion and cellular polarity, remodeling of the cytoskeleton, and changes in cell-matrix adhesion [63,64]. Epithelial cells convert into a mesenchymal phenotype not only during embryonic development, tissue regeneration, organ fibrosis, and wound healing but also in tumor progression with metastatic expansion, impacting on the resistance to cancer treatment. EMT is becoming a target of interest for anticancer therapy, and more knowledge about the role of EMT in metastasis, its control, and its reversion is necessary [63]. Snail, twist, and ZEB (Zinc finger E-box-binding homeobox 2) are the initiating transcription factors that drive the transition from epithelial to mesenchymal phenotype, affecting the expression of specific target genes; activating interstitial markers, including N-cadherin, vimentin, and fibronectin; or inhibiting epithelial markers (E-cadherin and β-catenin) [65]. Beyond the aforementioned transcription factors, a large number of lncRNAs, including pancRNAs, have been reported as regulators of the EMT process, regulating the transcription of their corresponding sense mRNAs.

For instance, the transcription of Vimentin (VIM) is positively correlated with the formation of a stable R-loop structure by a head-to-head antisense transcript (*VIM-AS1*). *VIM-AS1* decreases nucleosome occupancy and increases binding of transcription factors of the NF-κB (Nuclear Factor kappa-light-chain-enhancer of activated B cells) pathway on the promoter region, promoting the transcription of VIM gene. This activity is recognized as a general characteristic of GC-rich promoters with divergent sense/antisense transcription, as reported for *RPSAP52* (Ribosomal Protein SA Pseudogene 52) and its cognate gene *HMGA2* (High Mobility Group AT-Hook 2) [66]. Another important regulator of cancer invasiveness is the promoter-associated noncoding RNA *pancEts-1* [61]. As mentioned above, *pancEts-1* levels were positively correlated with those of ETS-1 in gastric cancer specimens, associated to poor outcome for patients [61]. *Panc-Ets1* promotes tumorigenesis and aggressiveness of gastric cancer cells interacting with NONO and favoring ERG-positive transcriptional activity on *ETS-1* gene [61]. *ETS-1* is a transcription factor found upregulated in many solid tumors and associated with tumor angiogenesis and metastasis. Indeed, in neuroblastoma cells, *pancEts-1* was shown to bind to heterogeneous nuclear ribo-nucleoprotein K (hnRNPK), increasing the stability and nuclear translocation of β-catenin, which, in turn, is able to activate the transcription of target genes and to facilitate anchorage-independent growth and invasiveness [67]. Similarly, the lncRNA *HIF2PUT* (hypoxia-inducible factor-2α (HIF-2α) promoter upstream transcript), the promoter-associated ncRNA of the *HIF-2α* gene, can inhibit proliferation, self-renewal, and migration of osteosarcoma stem cells by regulating the expression of *HIF-2α* [68].

Interestingly, two distinct transcripts, sense (S) and antisense (AS), have been found transcribed from the promoter region of E-cadherin gene (*CDH1*), generated from distinct initiation sites located upstream the *CDH1* TSS and displaying different characteristic and antagonistic effect on *CDH1* transcriptional regulation. The sense-directed ncRNAs *paRCDH1-S* is a chromatin-bound RNA that cooperates with AGO1 to recruit the Histone-lysine N-methyltransferase SUV39H1 to promote a repressive chromatin state in PC3 cells [69]. *paRCDH1-AS* is sensible to UHRF1-mediated regulation, as the *CDH1* host gene and acts as a scaffold for the epigenetic regulators UHRF1, DNMT3A, SUV39H1 and SUZ12 involved in *CDH1* repression, thus preventing their binding from the promoter region [70].

## 3. Mechanisms of Action of PancRNAs

By definition, pancRNAs are involved in the regulation of their host genes. However, the molecular mechanisms harnessed have not been completely elucidated yet. To date, multiple mechanisms have been proposed, leading either to the activation or repression of the host genes. The presence and strand orientation of pancRNAs do not represent a fixed feature but change dynamically depending on the cell type and experimental context and in relation to the transcriptional state of the neighboring genes [71].

**Figure 2:**
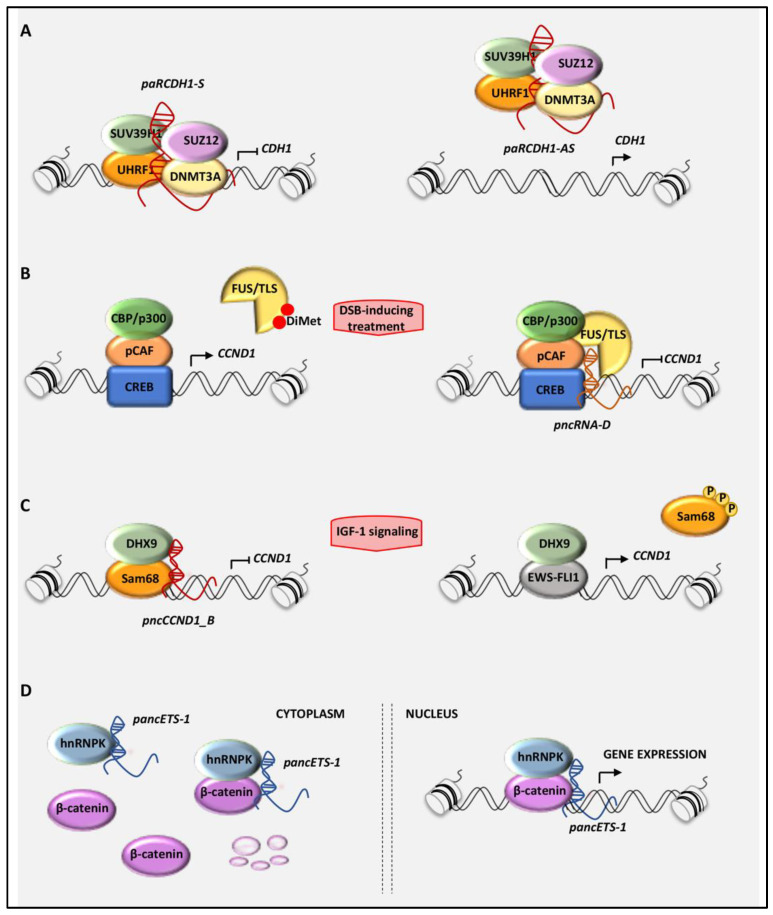
Schematic model of the molecular mechanisms leading to the gene transcriptional regulation mediated by pancRNAs: (**A**) The pancRNAs transcribed in sense orientation from the promoter of the *CDH1* gene (*paCDH-1S*) repress *CDH1* expression, whereas those antisense-directed (*paRCDH1-AS*) increase *CDH1* levels by preventing the repressive recruitment of specific epigenetic complex on the *CDH1* promoter. (**B**) *pncRNA_D* represses the transcription of *CCND1* by recruiting FUS/TLS (fused in sarcoma/translocated in liposarcoma) on the *CCND1* promoter, which in turn inhibits the histone acetyl-transferase (HAT) activity of CBP/p300. (**C**) In Ewing sarcoma cells, *pncCCND1_B* assembles a repressive complex formed by DHX9 and Sam68 on the *CCND1* promoter to repress *CCND1* transcription. On the contrary, the mitogenic stimulation with IGF-1 dissociates this complex, favoring the EWS-FLI1/DHX9-dependent transcription of *CCND1.* (**D**) In cancer cells, *pancEts-1* interacts with heterogeneous nuclear ribo-nucleoprotein K (hnRNPK), promoting the stabilization and transactivation of β-catenin.

Most active promoters are marked by strong enrichment of trimethylated H3K4 and depletion of monomethylated H3K4, together with acetylated H4, acetylated H3, and nucleosome depletion [12,72]. These chromatin features correlate with peaks of RNA polymerase II and TAF1 [72]. Transcription and recruitment of the pancRNAs can strongly affect promoter features. Initially, pancRNAs have been proposed as key effectors in the epigenetic silencing of nearby genes, although later on, genome-wide studies demonstrated a positive effect of pancRNAs toward the neighboring host genes [30,32]. To date, the emerging picture depends on the features imposed by the local chromatin conformation, suggesting both enhancing and repressive functions. In specific cases, the concurrent transcription of both sense and antisense pancRNAs from the same promoter has been shown, as for *CDH1* [70,71]. The sense-directed ncRNAs arising from this promoter region mediate *CDH1* repression, whereas antisense-directed ncRNAs (*paRCDH1-AS*) are necessary for its expression [70]. Mechanistically, *paRCDH1-AS* recruits epigenetic regulators, including UHRF1, DNMT3A, SUV39H1, and SUZ12, to prevent their repressive recruitment on the *CDH1* promoter (Figure 2A). Thus, knockdown of *paRCDH1-AS* leads to *CDH1* repression by switching the chromatin engagement of the repressive complex.

As mentioned above, to accomplish their function, pancRNAs often interact with RNA-binding proteins (RBPs), that modulate their activity by shaping the recruited molecular complex. As an example, the *pncRNA-D* transcribed from the promoter of the *CCND1* gene was shown to recruit the RBP FUS/TLS and CBP/p300, leading to the inhibition of HAT activity and to the repression of *CCND1* (Figure 2B). Abolishment of *pncRNA-D* binding caused conformational remodeling in the FUS/TLS protein, reducing the affinity to CBP/p300 [26]. Notably, arginine methylation of FUS/TLS abrogates FUS/TLS-mediated repression of CBP/p300 HAT activities. Cui and colleagues [26] show that PRMT1 (Protein Arginine Methyltransferase 1) catalyzes di-methylation on the Arg-476 of FUS/TLS; this posttranslational modification inhibits FUS/TLS binding to RNA, thus inducing the release of the CBP/p300 HAT activity from the FUS/TLS driven inhibition. FUS/TLS belongs to the FET family of RBPs [73] deeply involved in human disease, including motor neuron disorders and cancer [23,73]. In Ewing sarcoma, the FUS/TLS gene gives rise to chromosomal translocations involved in the transformation process [73]. Thus, the possibility to interfere with the RNA-binding properties of FUS/TLS could be further exploited for therapeutic purpose.

Similarly to FUS/TLS, the RBP Sam68 inhibits *CCND1* transcription by forming a molecular complex with DHX9 and *pncCCND1_B* [22]. In Ewing sarcoma cells, two mutually exclusive complexes occur, formed by DHX9 with either EWS-FLI1 or Sam68/*pncCCND1_B* [22]. A switch between the two complexes can be promoted by the IGF-1 signaling pathway, which is a critical contributor to malignant transformation and chemotherapy resistance of Ewing sarcoma [74]. Notably, IGF-1 signaling inhibits the formation of the DHX9–Sam68 complex by affecting tyrosine phosphorylation of Sam68. Tyrosine phosphorylation is known to lower the affinity of Sam68 for RNA [32,75], and IGF-1-induced activation of SRC promotes Sam68 phosphorylation and the release of the *pncCCND1_B*. Thus, tyrosine phosphorylation of Sam68 upon mitogenic stimuli mimics Sam68 silencing by impairing the ability of *pncCCND1_B* to repress the *CCND1* promoter, leading to the upregulation of cyclin D1 expression (Figure 2C) [22]. The emerging hypothesis is that multiple transcripts driven by the same promoter can synergistically or antagonistically work to promote or repress gene expression. Importantly, the activity and recruitment of these pancRNAs is strictly dependent on the regulatory features of interacting RBPs.

In a similar fashion, *pancEts-1* directly interacts with two different RBPs, hnRNPK and NONO, in cancer cells [61,67]. In neuroblastoma cells, the direct binding of *panc-Ets-1* to hnRNPK favors the interaction with β-catenin, thus resulting in β-catenin stabilization and transactivation, to assist tumor invasion and metastasis (Figure 2D) [67]. hnRNPK is an evolutionarily conserved nucleocytoplasmic shuttling protein that participates in several aspects of RNA metabolism, including transcription, translation, mRNA splicing, mRNA stability, and chromatin remodeling [76]. In gastric cancer, the interaction of pancEts-1 with NONO and ERG promotes ERG transactivation and upregulation of ETS-1 expression, which contributes to aberrant proliferation and invasiveness of gastric cancer cells [67]. *PancEts-1* binds the RNA recognition motif 1 (RRM1) domain of NONO protein and exerts its oncogenic function, at least in part, by modulating NONO activity [62].

Thus, pancRNAs orchestrate gene expression by intervening in different biological aspects, ranging from promoter regulation and transcriptional control to chromatin structure arrangement. The comprehension of these molecular mechanisms might provide novel insights into fundamental aspects of gene regulation.

## 4. Therapeutic Targeting: Lessons from PancRNAs

Mounting evidence indicates that lncRNAs are promising biomarkers in the diagnosis and prognosis of cancer, especially their presence in body fluids. These findings highlight their diagnostic and prognostic potentials and open the path for novel therapeutic strategies [77].

As widely discussed in the previous sections, pancRNAs modulate gene expression through several mechanistic strategies. Nevertheless, their functional role is strictly confined to the promoter regions where they are embedded, thus providing specificity for the modulation of their host gene. The ability to selectively activate or inhibit gene expression is fundamental to understanding cellular systems and developing therapeutics. In line with this, pancRNAs transcribed from the promoter of genes involved in cancer could represent alternative molecular candidates for RNA-based anticancer strategies. The use of small interference RNA (siRNA) is becoming an emerging tool for its therapeutic potential [78]. In vitro siRNA transfection can efficiently reduce the pancRNA transcribed from the elongation factor 1 alpha (*EF1α*) promoter. Interestingly, degradation of this pancRNA by antisense phosphorothioate oligodeoxynucleotides (ODN) exhibited high specificity, since it did not affect *EF1α* mRNA transcript [20]. This work highlighted the relevance of these low copy number transcripts in the modulation of the transcriptional gene silencing; the same strategy was also efficiently applied to interfere with a promoter-associated lncRNA of the *MYC* gene, leading to profound inhibition of the development of prostate tumor xenografts [37,79]. Another evidence of the potential relevance of targeting pancRNAs in cancer therapy comes from the work performed by the laboratory of Dr. Grummt [44,80]. Transfection of a short synthetic RNA comprising the triplex-forming region of the pancRNAs *Khps1* led to impaired cell migration, invasion, and clonogenicity of cancer cells. As mentioned above, *Khps1* is synthesized in antisense orientation to the proto-oncogene *SPHK1* and is required for the activation of *SPHK1* transcription by establishing a transcription-permissive chromatin structure and by increasing CBP/p300 occupancy and H3K27 acetylation to ensure E2F1 binding [44,80]. The discovery of this regulatory loop not only suggests the potential of using the pancRNA *Khps1* as a biomarker but also represents a promising step toward therapeutic intervention.

Similar to the pancRNA molecular proceeding, multiple strategies have been designed. Several groups reported that targeting promoter regions with dsRNAs was effective in inducing transcriptional silencing by triggering histone modification and/or DNA methylation. This method was successfully used to downregulate CDH1 expression in breast cancer cells [81]. Notably, analysis of the promoter chromatin feature after dsRNA treatment revealed a general enrichment of H3 dimethyl-K4, a histone mark associated with actively transcribed promoters, and H3 dimethyl-K9 residues, a modification present at inactive promoters, only at the targeted *CDH1* promoter [81]. A similar approach was also successfully applied to downregulate the human RASSF1A (Ras association domain family 1 isoform A) gene, encoding a putative tumor suppressor that is hypermethylated in a variety of human cancers. ShRNAs (Short hairpin RNAs) complementary to the *RASSF1A* promoter increase de novo DNA methylation and gene silencing [82]. These reports highlight the possibility to selectively target gene expression at specific promoters.

PancRNAs can either repress or activate gene expression of their host genes. Likewise, small double-stranded RNA (dsRNA) molecules were developed to either repress or activate endogenous gene transcription. These last molecules were termed small activating RNA (saRNA) and consist of 21-nt dsRNAs targeting selected promoter regions of human genes such as p21WAF1/CIP1 (p21), E-cadherin, and VEGF (Vascular Endothelial Growth Factor). SaRNAs were able to induce an increase in mRNA and protein levels when transfected in vitro [83]. SaRNA targeting the p21 gene promoter region could induce cell-cycle arrest and apoptosis via upregulation of the expression of p21 in human prostate and bladder cancer cells [84,85]. Mechanistically, the dsRNA-induced gene activation requires the AGO2 protein and is associated with a loss of lysine-9 methylation on histone 3 at target sites. Moreover, saRNA can associate specifically with the RBP hnRNPA2/B1. Notably, knockdown of hnRNPA2/B1 blocks the saRNA-mediated p21 induction [86]. These findings reveal the pivotal role of RBP in this process: in fact, hnRNPA2/B1 facilitates recognition of the p21-specific promoter by the saRNA [86].

Similarly, saRNA mediating upregulation of the endogenous E-cadherin reduced cell proliferation, promoted apoptosis, decreased mobility, and inhibited tumor growth in breast cancer xenograft mice [87]. Therapeutic potential of this approach has been investigated also by intravesical delivery to facilitate p21 induction, with promising results showing extended animal survival and inhibited tumor growth in mice with orthotopic bladder cancer [88].

Collectively, these reports highlight the potential of therapeutic use of dsRNA in targeting gene activation or repression at promoters, analogously to pancRNA proceeding. Moreover, saRNA could be further applied in combination with other drugs to improve treatment efficacy. Indeed, saRNAs targeting p21 enhance the sensitivity of A549 non-small-cell lung carcinoma to cisplatin [89]. Overall, pancRNA-based regulatory circuits might be further exploited to develop novel therapeutic strategies.

## 5. Conclusions

During the last decade, considerable developments have been achieved in understanding the regulatory role of pancRNAs in physiological and pathological processes. Gene activation or repression by pancRNAs finely shape tissue-specific patterns but can also contribute to the transformation process. Recent advances showed that pancRNAs are targetable molecules and that modulation of their expression can be used for therapeutic purpose. Notably, pancRNA-mediated epigenetic dynamics can be mimicked by RNA oligonucleotides, exploitable for therapeutic purpose.

To this regard, several therapeutic oligonucleotides have been recently developed for clinical use [90,91,92,93]. This shows that small dsRNA molecules can be employed to treat diseases previously undruggable, making reasonable to consider ncRNA-based approaches as suitable and efficacious strategies even for cancer treatment.
